# Molecular mechanisms of esophageal epithelial regeneration following repair of surgical defects with acellular silk fibroin grafts

**DOI:** 10.1038/s41598-021-86511-9

**Published:** 2021-03-29

**Authors:** Gokhan Gundogdu, Mehmet Tosun, Duncan Morhardt, Ali Hashemi Gheinani, Khalid Algarrahi, Xuehui Yang, Kyle Costa, Cinthia Galvez Alegria, Rosalyn M. Adam, Wei Yang, Joshua R. Mauney

**Affiliations:** 1 Departments of Urology and Biomedical Engineering, University of California, Irvine, Orange, CA 92868 USA; 2 Urological Diseases Research Center, Boston Children’s Hospital, Boston, MA 02115 USA; 3 Department of Surgery, Harvard Medical School, Boston, MA 02115 USA; 4 Broad Institute of the Massachusetts Institute of Technology and Harvard University, Cambridge, MA 02142 USA; 5 Division of Cancer Biology and Therapeutics, Departments of Surgery and Biomedical Sciences, Samuel Oschin Comprehensive Cancer Institute, Cedars-Sinai Medical Center, Los Angeles, CA 90048 USA; 6 Department of Medicine, David Geffen School of Medicine, University of California, Los Angeles, Los Angeles, CA 90095 USA; 7 Departments of Urology and Biomedical Engineering, University of California, Irvine, Building 55, 101 The City Drive South., Rm. 300, Orange, CA 92868 USA

**Keywords:** Biological techniques, Cell biology, Gastroenterology

## Abstract

Constructive remodeling of focal esophageal defects with biodegradable acellular grafts relies on the ability of host progenitor cell populations to repopulate implant regions and facilitate growth of de novo functional tissue. Intrinsic molecular mechanisms governing esophageal repair processes following biomaterial-based, surgical reconstruction is largely unknown. In the present study, we utilized mass spectrometry-based quantitative proteomics and in silico pathway evaluations to identify signaling cascades which were significantly activated during neoepithelial formation in a Sprague Dawley rat model of onlay esophagoplasty with acellular silk fibroin scaffolds. Pharmacologic inhibitor and rescue experiments revealed that epithelialization of neotissues is significantly dependent in part on pro-survival stimuli capable of suppressing caspase activity in epithelial progenitors via activation of hepatocyte growth factor receptor (c-MET), tropomyosin receptor kinase A (TrkA), phosphoinositide 3-kinase (PI3K), and protein kinase B (Akt) signaling mechanisms. These data highlight the molecular machinery involved in esophageal epithelial regeneration following surgical repair with acellular implants.

## Introduction

Surgical management of full thickness, esophageal defects is often necessary during repair of benign refractory strictures as well as in situations where tissue loss has occurred as a result of perforation or tumor resection^1–4^. Onlay esophagoplasty with autologous tissue flaps is utilized as the primary clinical approach to reconstruct focal esophageal injuries and reestablish organ continuity. However, drawbacks with this strategy including donor site morbidity and restricted tissue availability have spurred research into alternative treatment modalities^5,6^. Acellular matrices derived from decellularized tissues, synthetic polymers, or silk fibroin (SF) represent emerging technologies designed to overcome limitations associated with the use of autologous tissues by serving as “off-the-shelf” scaffolds for esophageal tissue engineering^7–9^. These biomaterials have been demonstrated to support esophageal defect consolidation in a variety of preclinical animal models^7–9^ and in some cases short-term human trials^10^. Cell-free scaffolds rely on host progenitor cell populations to repopulate implant microenvironments and facilitate growth of de novo functional tissue. To date, intrinsic molecular mechanisms governing these repair processes remain poorly understood. Delineation of signaling pathways with key roles in scaffold-mediated, constructive remodeling may lead to advancements in instructive biomaterial design as well as elucidate new therapeutic targets to enhance wound healing.

The mammalian esophagus is lined by a multi-layered, stratified squamous epithelium which serves to protect underlying tissue from food bolus abrasion, luminal acidity, and invasive pathogens^11,12^. Homeostasis of the esophageal epithelium is mediated by a basal cell progenitor population which lines the basement membrane and divides stochastically to produce proliferating and differentiating daughter cells with equal probability^13,14^. Upon injury, basal cell progenitors undergo reversible fate switching and produce an excess of proliferating daughter cells that then migrate into the defect site and differentiate to restore native tissue architecture^14^. In the present report, we employed mass spectrometry-based quantitative proteomics and in silico pathway analysis to identify signal transduction clusters enriched during neoepithelial formation following onlay esophagoplasty in rats. Pharmacologic inhibitor and rescue experiments demonstrated that epithelialization of neotissues is dependent in part on pro-survival stimuli capable of suppressing caspase activity in epithelial progenitors via activation of hepatocyte growth factor receptor (c-MET), tropomyosin receptor kinase A (TrkA), phosphoinositide 3-kinase (PI3K), and protein kinase B (Akt) signaling mechanisms. These findings shed light on the molecular machinery involved in esophageal epithelial regeneration following surgical reconstruction.

## Results

**Figure 1:**
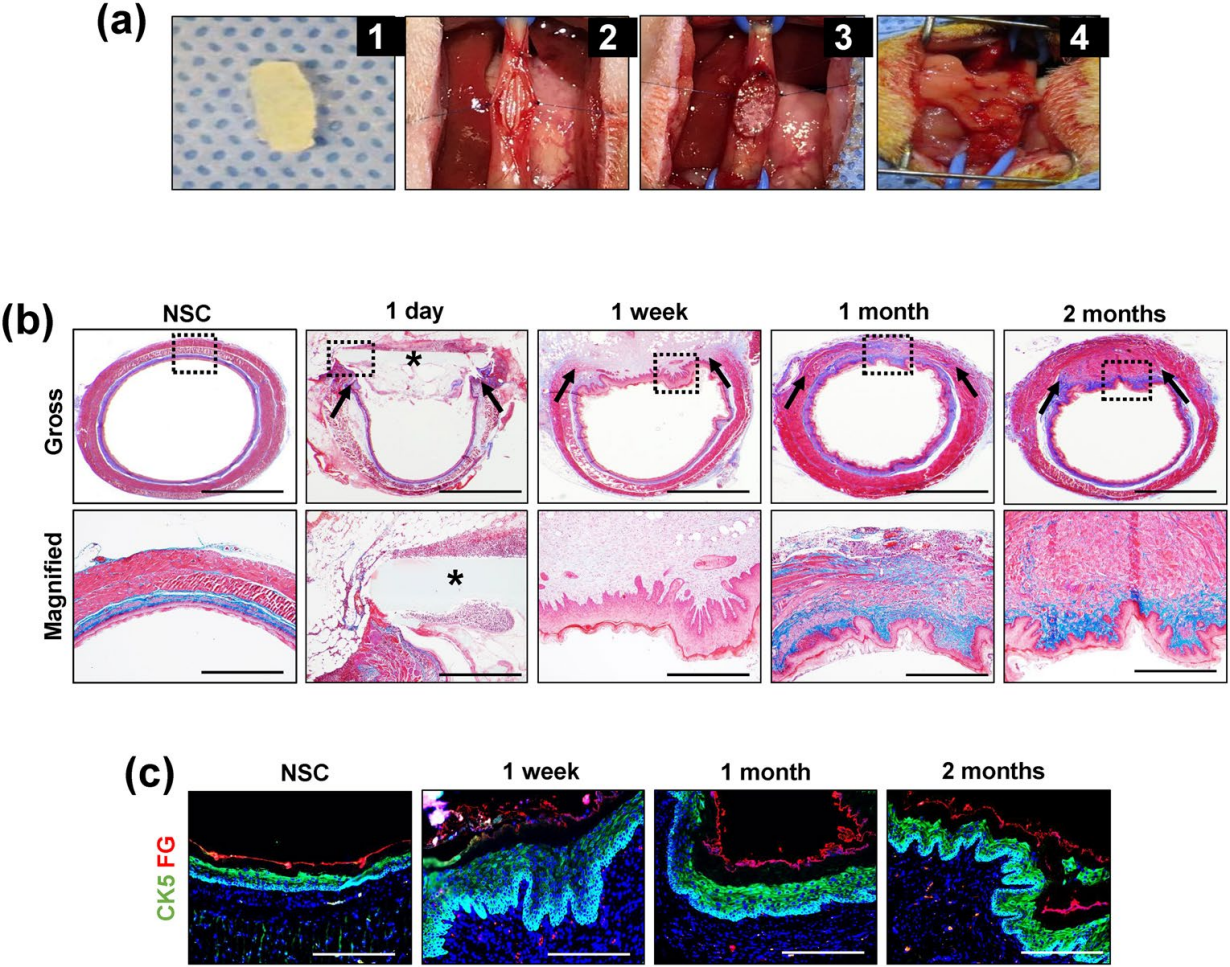
Onlay esophagoplasty model and histological and immunohistochemical analyses of constructive remodeling at implant sites. (**a**) Photomicrographs of various surgical stages of BLSF graft implantation into the rat esophagus. (**a1**) BLSF scaffold (7 × 3 mm) prior to implantation. (**a2**) Excision of native tissue and patch defect creation. (**a3**) Anastomosis of BLSF graft (S) into the esophageal defect. (**a4**) Implant area covered with omental patch tethered to esophageal adventitia prior to abdominal and skin closure. (**b**) Representative photomicrographs of gross and magnified (bracketed fields in top row) esophageal cross-sections stained with Masson’s trichrome from nonsurgical controls (NSC) and rats subjected to onlay esophagoplasty with acellular grafts and harvested at study timepoints. Scale bars = 1.25 mm (top row) and 750 μm (bottom row). Asterisks denote scaffold fragments and arrows demarcate anastomotic borders of the original implantation site. (**c**) Representative photomicrographs of epithelia in NSC and neotissues supported by acellular grafts demonstrating protein expression of cytokeratin (CK)5 and filaggrin (FG). For all panels, respective marker expression is displayed in red (FG) or green (CK5) while DAPI nuclear counterstain is detailed in blue. Cyan coloring is a consequence of merging CK5 and DAPI staining. Scale bars = 200 µm.

The main goals of our study were to (1) establish a temporal profile of the signaling cascades that occur during scaffold-mediated, constructive remodeling of the rat esophagus and (2) determine the significance and function of these pathways in neoepithelial formation at graft sites. The survival rate for onlay esophagoplasty procedures with BLSF grafts was 94% with 6% mortality observed (8/136 rats) from anesthesia-related complications (2 animals), abdominal wound dehiscence (1 animal), and esophageal obstruction secondary to fur ingestion (5 animals). Histological, IHC, and histomorphometric evaluations were first performed to characterize the various stages of tissue regeneration and host tissue responses in our rat model of onlay esophagoplasty with BLSF scaffolds (Fig. 1). One day after esophageal reconstruction, mononuclear inflammatory cells and neutrophils were detected throughout the implant area with evidence of initial biomaterial fragmentation observed. By 1 week post-op, the BLSF graft had markedly degraded and the implant site was occupied by a fibrovascular scar populated by mononuclear inflammatory cells and myofibroblasts. Neotissues were lined by a stratified squamous, keratinized epithelium consisting of Krt5 + basal and suprabasal cells as well as FG + superficial cell layers. At this timepoint, the neoepithelium was hyperplastic and contained proliferating Ki67 + pan-CK + cells. Following 1 month of scaffold implantation, an extracellular matrix rich lamina propria had developed and both host skeletal and smooth muscle bundles could be seen infiltrating the graft region. Further maturation of the de novo muscularis mucosa and muscularis externa was observed at 2 months as indicated by qualitative increases in muscle density within the central graft area in respect to early timepoints. Epithelial hyperplasia in neotissues was qualitatively reduced at both 1 and 2 months in respect to 1 week of wound healing. These data demonstrate that BLSF grafts support constructive remodeling of rat esophageal defects and de novo epithelial formation occurs within the first week of scaffold implantation.

**Figure 2:**
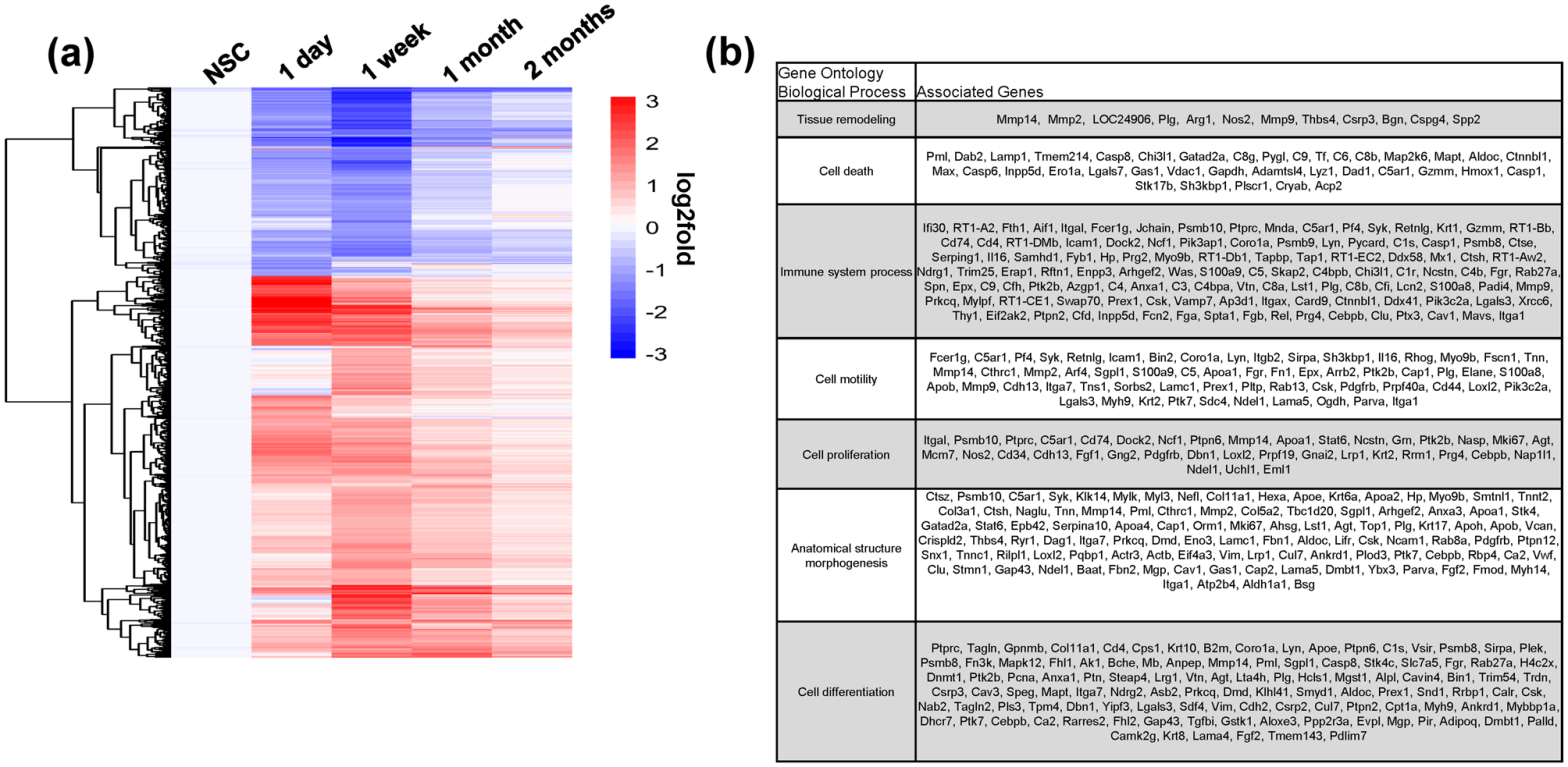
Temporal kinetics and gene ontology analyses of protein species present in neotissues over the course of scaffold-mediated, tissue regeneration. (**a**) Heatmap of 1,506 proteins found regulated by twofold (log2ratio > 1 or <  − 1) in at least one reconstructed group (1 day, 1 week, 1 month, 2 months post-op) relative to NSC controls. The heatmap was generated using pHeatmap package (v1.0.12) in RStudio (v1.3.1093) detailed at http://www.rstudio.com/. (**b**) Gene ontology analysis of proteins displayed in (**a**) with biological functions relevant to regenerative processes.

Quantitative proteomic analysis of the esophageal tissues identified a total of 5682 proteins with a false discovery rate of 1%. Among these, 4,150 proteins were quantified across all the 20 tissue samples (5 time points × 4 replicates) and used for statistical analysis. A total of 1506 proteins were found to be regulated by > twofold (log2ratio > 1 or <  − 1) in at least one reconstructed group (1 day, 1 week, 1 month, 2 months post-op) relative to NSC controls (Supplementary Table S1, Fig. 2a). Gene Ontology (GO) enrichment evaluations revealed subgroups of genes coding for these proteins with biological functions associated with wound healing processes including tissue remodeling, cell death, immune system process, cell motility, cell proliferation, anatomical structure morphogenesis (Fig. 2b).

**Figure 3:**
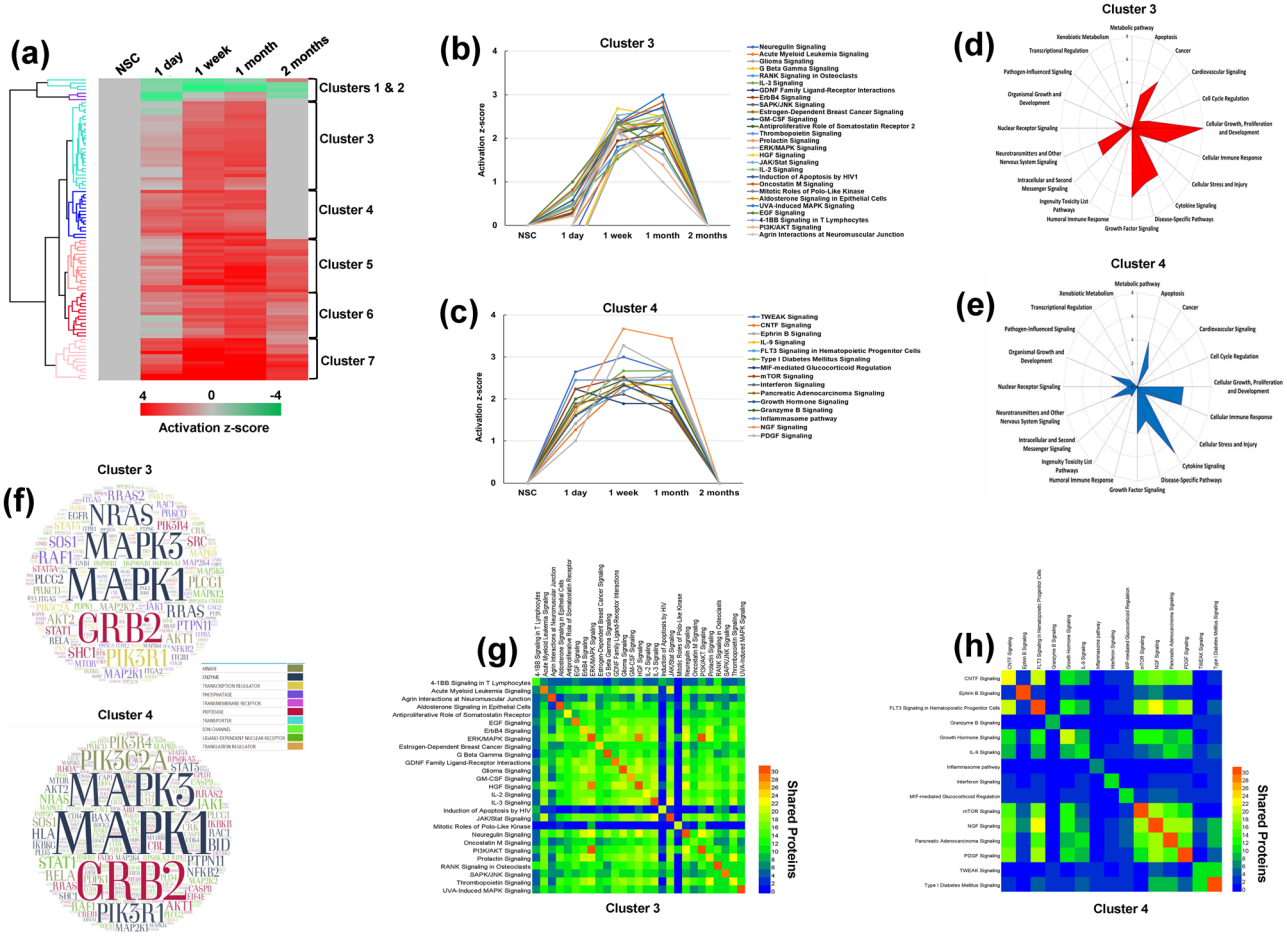
Temporal proteomic and in silico analyses of signaling pathway modulation in rat esophageal tissues following reconstruction with acellular grafts. (**a**) Heatmap and unbiased clustering of signaling cascades based on z-score activation over the course of esophageal healing. N = 4 animals were analyzed per experimental condition. Each column represents pathway status of NSC or neotissues at experimental timepoints. Red = activated, Green = inhibited, Grey = baseline normalized to nonsurgical controls (NSC). The heatmap was generated using pHeatmap package (v1.0.12) in RStudio (v1.3.1093) detailed at http://www.rstudio.com/. (**b,c**) Line charts of activation z-scores of pathways contained in Clusters 3 (**b**) and 4 (**c**). (**d,e**) Radar graphs of significantly activated pathways Clusters 3 (**d**) and 4 (**e**) demonstrating biological functions. (**f**) Word clouds showing the most recurrent proteins and their functions in pathways identified in Clusters 3 [top plot] and 4 [bottom plot]. (**g,h**) Heatmaps showing the level of internal connectivity (shared proteins) between pathways in Clusters 3 (**G**) and 4 (**h**).

Significantly enriched canonical pathways were identified from in silico analyses of DEP present at various stages of neotissue maturation. Unsupervised hierarchical clustering of predicted pathways was performed based on their temporal activation pattern and revealed two main superclusters (Fig. 3a). The first supercluster consisted of two subclusters (Clusters 1 and 2), each with pathways which were inhibited in host tissue following injury and, in most cases remained attenuated in neotissues relative to NSC for the entire study period (Supplementary Fig. S1). The second supercluster was comprised of five subclusters (Clusters 3–7) with each containing signaling cascades activated in neotissues by 1 week post-op over NSC levels. The activation status of pathways in Clusters 3 and 4 returned to baseline in neotissues 2 months following esophageal reconstruction (Fig. 3b,c), while Clusters 5–7 remained active for the duration of the experiment (Supplementary Fig. S1). Overall, our results show that scaffold-mediated, esophageal wound healing is associated with both repression and activation of selective molecular signaling patterns.

We chose to further explore the biological function of pathways in Clusters 3 and 4 for potential regulators of epithelial regeneration since the bulk of their activation profiles peaked during neoepithelium formation. Biological pattern analysis of significantly regulated pathways in Cluster 3 indicated the involvement of 7 pathways in cellular growth and proliferation, 5 pathways in growth factor signaling, 4 pathways in cytokine signaling, and 3 signaling cascades in apoptosis (Fig. 3d). On the other hand, Cluster 4 revealed 4 pathways associated with cellular growth and proliferation and 6 pathways implicated in cytokine signaling (Fig. 3e). The frequency of protein species present in pathways from Clusters 3 and 4 were also identified using the Word Cloud tool (Fig. 3f). In both clusters, enzymes such as mitogen-activated protein kinase 1 (MAPK1), MAPK3, and the adaptor protein, growth factor receptor-bound protein 2 (GRB2) had the highest degree of enrichment. The abundance of these factors during the regenerative process may suggest their involvement in a crucial regulatory node, however further experimentation is needed. The total number of proteins shared between pathways in each individual cluster revealed that signaling cascades in Cluster 3 were generally more integrated with each other (Fig. 3g) in comparison to Cluster 4 which showed less synergy between pathway members (Fig. 3h). Overall, in silico analyses of Clusters 3 and 4 predicted that signaling pathways governing cellular growth and proliferation as well as cytokine signaling are significantly activated during de novo epithelial formation.

**Figure 4:**
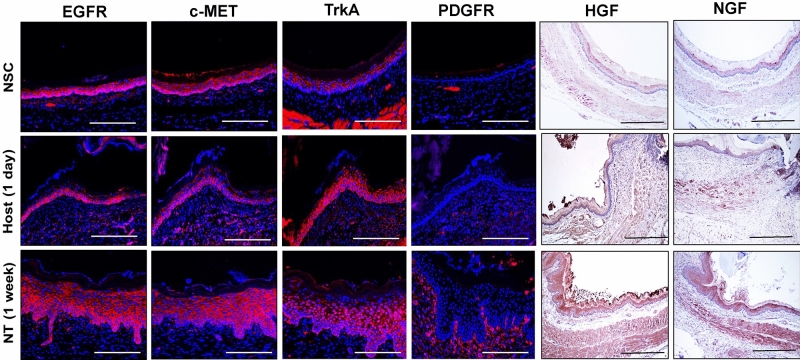
Expression of selective receptor tyrosine kinases and their associated ligands in control and neotissues. Representative photomicrographs of mucosa in nonsurgical controls (NSC), host-graft interface at 1 day post-op, and in neotissues (NT) at 7 days following graft implantation. For columns 1–4, respective marker expression is displayed in red with DAPI nuclear counterstain in blue. For columns 5 and 6, respective marker expression is denoted in brown (HRP labeling) with hematoxylin nuclear counterstain in blue. Scale bars for all panels = 200 µm.

**Figure 5:**
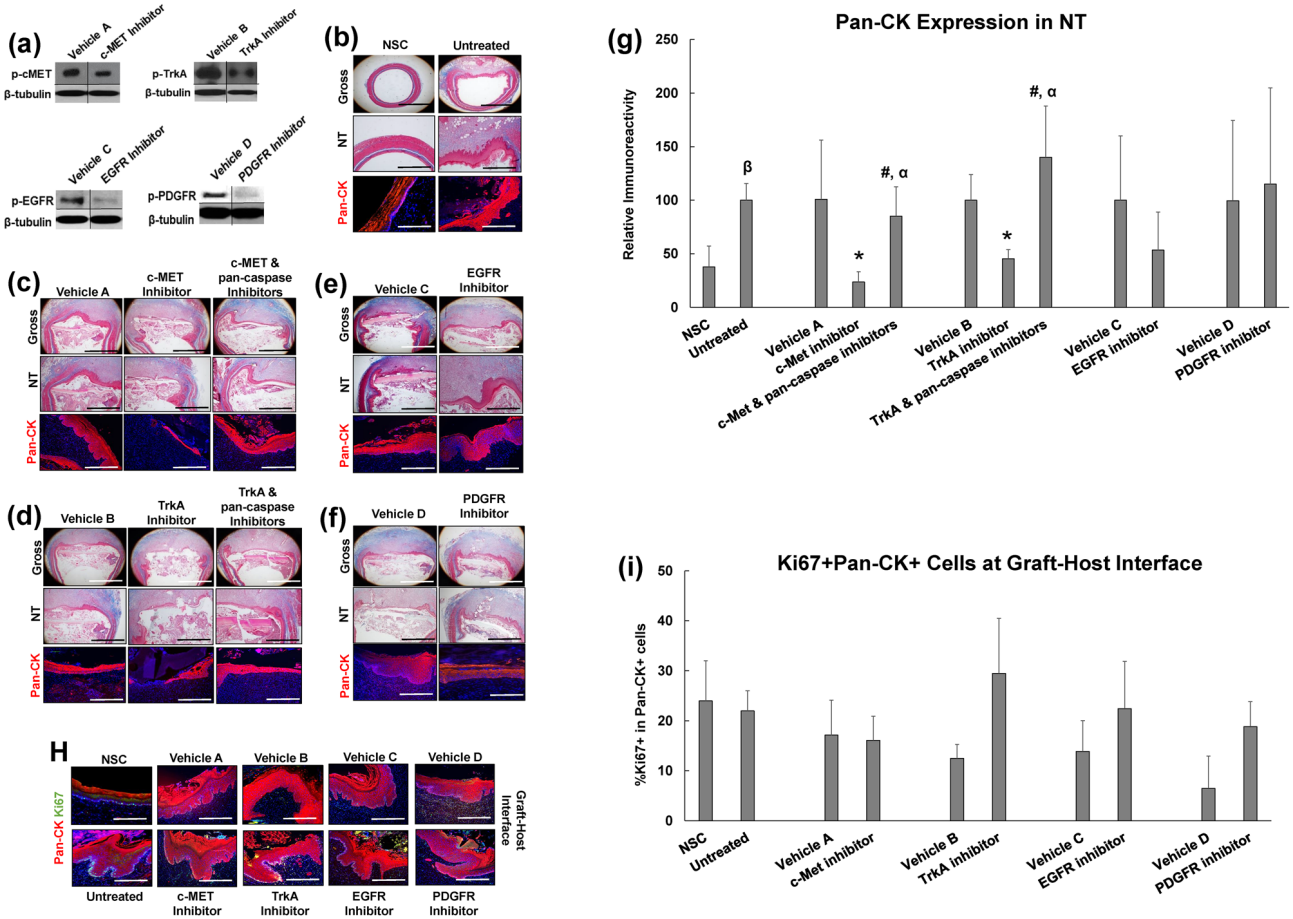
The role of selective receptor tyrosine kinase activation in esophageal epithelial regeneration. (**a**) Immunoblot analyses of p-cMET, p-TrkA, p-EGFR, p-PDGFR and β-tubulin protein levels from neotissues treated with vehicle or inhibitors for 7 days of scaffold implantation. Vehicle and inhibitor comparisons were made on the same immunoblots and with equal amounts of protein loaded per lane (N = 3–4 animals per group). Targeted bands were cropped from original full length immunoblots displayed in Supplementary Fig. S2. (**b**–**f**) Representative photomicrographs of global and magnified esophageal cross-sections from nonsurgical controls (NSC) and untreated surgical controls as well as neotissues (NT) treated with vehicles or RTK inhibitors with or without pan-caspase inhibition for 7 days post-op. Samples were stained with Masson’s trichrome (top 2 rows in each panel) or subjected to immunohistochemical analysis for pan-cytokeratin (CK) expression (bottom row in each panel). Scale bars for top, middle, and bottom rows are respectively 1.25 mm, 500 μm, and 200 μm. (**h**) Immunohistochemical evaluations of Ki67 + pan-CK + epithelial cells in NSC and host-graft interface in specimens described in (**b**–**f**). Scale bars in (**h**) for all photomicrographs are 200 μm. For (**b**–**h**), respective pan-CK expression is displayed in red, Ki67 expression is displayed in green and blue represents DAPI nuclear counterstain. (**g, i**) Histomorphometric evaluations of pan-CK expression in controls and neotissues described in (**b**–**f**) as well as the percentage of Ki67 + pan-CK + epithelial cells at the host-graft interface in samples described in (**h**). N = 4–8 rats per group and 2–4 sections per animal were analyzed for each data point. For (**g**,**i**), multi-group (> 3 groups) comparisons were performed with the Kruskal Wallis test in combination with the post hoc Dunn’s test. Two group comparisons were performed with the Mann–Whitney U test. (*) = p < 0.05 in comparison to respective vehicle control. (#) = p < 0.05 in comparison to respective inhibitor group. (α) = p > 0.05 in comparison to respective vehicle cohort. (β) = p < 0.05 in comparison to NSC. Data are expressed as means ± standard deviations.

Several cytokine signaling pathways in Clusters 3 and 4 including EGF, NGF, PDGF, and HGF signaling have been previously implicated in esophageal epithelial regeneration^15–18^. However, the significance of these molecular mechanisms in regulating the repair of implant sites is unknown. We first sought to determine the expression pattern of receptor tyrosine kinases (RTKs) governing activation of these pathways as well as their associated ligands prior to surgery and during neotissue maturation (Fig. 4). Upon selective ligand binding, RTKs located on the plasma membrane regulate activation of growth factor/cytokine signaling by transmitting downstream intracellular messengers capable of influencing diverse biological processes^19^. IHC analyses revealed EGFR (EGF signaling), TrkA (NGF signaling), and c-MET (HGF signaling) expression were localized to basal and suprabasal cell populations in both NSC and regenerating tissues. In addition, respective c-MET and TrkA ligands, HGF and NGF, were detected in the epithelia and underlying mucosa in the NSC cohort with qualitative increases observed in neotissues by 7 days post-operatively. PDGFR expression was scant in NSC, but was enriched in fibroblasts and vascular smooth muscle cells throughout the mucosa of neotissues by 7 days post-op. Phosphorylation of c-MET, TrkA, EGFR, and PDGFR was substantially mitigated in the presence of pathway-specific inhibitors in comparison to relative vehicle controls (Fig. 5a). Interestingly, pharmacologic inhibition of TrkA and c-MET activation led to significant attenuation of pan-CK + epithelial formation at implant sites, while EGFR and PDGFR antagonists had no significant effect in respect to vehicle controls (Fig. 5b–g). These data demonstrate that activation of both HGF/c-MET and NGF/TrkA signaling is required to reestablish epithelial integrity after biomaterial implantation.

Next, we investigated how HGF and NGF signaling cascades influence epithelialization of reconstructed esophageal tissues. Previous reports have shown that these molecular pathways can modulate various cellular processes important for wound healing including proliferation, migration, differentiation and survival^20,21^. IHC and histomorphometric analyses of Ki67 + pan-CK + epithelial cells at the hyperplastic, host-graft interface revealed no significant difference between any inhibitor-treated group and their corresponding controls (Fig. 5h,I). These observations suggest that HGF and NGF pathways influence epithelial regeneration in a manner independent of cell cycle regulation. The relevance of pro-survival functions of HGF and NGF signaling cascades in esophageal regeneration was determined by administration of c-MET or TrkA antagonists in combination with the pan-caspase inhibitor, Z-VAD-fmk in rats subjected to surgical repair (Fig. 5C,D,G). Over the course of 7 days of graft implantation, inhibition of pan-caspase activity fully rescued epithelial deficiencies encountered in groups treated with c-MET and TrkA antagonists alone. These results provide evidence that HGF/c-MET and NGF/TrkA signaling control epithelial regeneration in neotissues via inhibition of caspase activity in epithelial progenitors.

**Figure 6:**
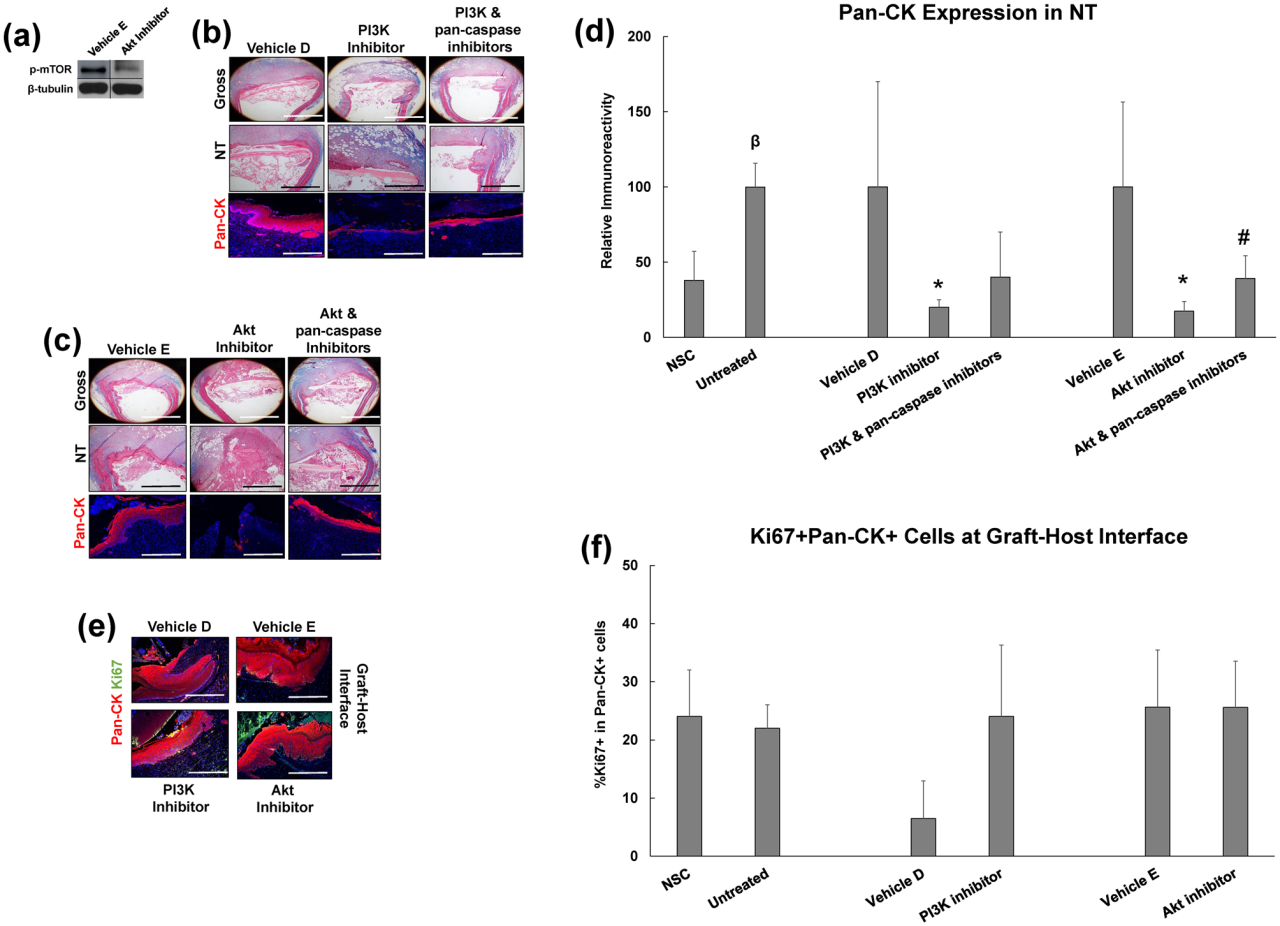
The role of secondary signaling messengers in constructive remodeling of esophageal tissue defects. (**a**) Immunoblot analysis of Akt target, p-mTOR and β-tubulin protein levels from neotissues treated with vehicle or Akt inhibitor for 7 days of scaffold implantation. Vehicle and inhibitor comparisons were made on the same immunoblots and with equal amounts of protein loaded per lane (N = 3–4 animals per group). Targeted bands were cropped from original full length immunoblots displayed in Supplementary Fig. S2. (**b**,**c**) Representative images of global and magnified esophageal cross-sections from neotissues (NT) treated with vehicles or RTK inhibitors with or without pan-caspase inhibition for 7 days post-op. Specimens were stained with Masson’s trichrome (top 2 rows in each panel) or subjected to immunohistochemical analysis for pan-cytokeratin (CK) expression (bottom row in each panel). Scale bars for top, middle, and bottom rows are respectively 1.25 mm, 500 μm, and 200 μm. (**e**) Immunohistochemical evaluations of Ki67 + pan-CK + epithelial cells at the host-graft interface in specimens described in (**b**,**c**). Scale bars in (**h**) for all photomicrographs are 200 μm. For (**b**–**h**), respective pan-CK expression is displayed in red, Ki67 expression is displayed in green and blue represents DAPI nuclear counterstain. Scale bars in (**e**) for all photomicrographs are 200 μm. For (**b**,**c**,**e**), respective pan-CK expression is displayed in red, Ki67 expression is displayed in green and blue represents DAPI nuclear counterstain. (**d,f**) Histomorphometric evaluations of pan-CK expression in NSC, untreated controls and neotissues described in (**b**,**c**) as well as the percentage of Ki67 + pan-CK + epithelial cells at the host-graft interface in samples described in (**e**). N = 4–8 rats per group and 2–4 sections per animal were analyzed for each data point. For panels (**d**,**f**), multi-group (> 3 groups) comparisons were performed with the Kruskal Wallis test in combination with the post hoc Dunn’s test. Two group comparisons were performed with the Mann–Whitney U test. (*) = p < 0.05 in comparison to respective vehicle control. (#) = p < 0.05 in comparison to respective inhibitor group. (β) = p < 0.05 in comparison to NSC. Data are expressed as means ± standard deviations.

Published studies in a variety of in vitro and in vivo model systems have shown that c-MET and TrkA receptor activation leads to phosphorylation of several effectors including the PI3K/Akt signaling cascade which can mediate pro-survival functions^22,23^. Our proteomic analysis also revealed that Cluster 3 signaling pathways governed by PI3K/Akt signaling had a peak activation z-score during de novo epithelial formation. Therefore, we performed selective inhibition of the PI3K/Akt pathway in order to determine its role in epithelial regeneration following onlay esophagoplasty (Fig. 6). We first analyzed the effects of the Akt inhibitor, GSK690693 on the phosphorylation of the downstream Akt target, m-TOR since GSK690693 is known to upregulate Akt phosphorylation via a feedback loop, but not its downstream effectors^24,25^. Our immunoblot results demonstrated that following 7 days of GSK690693 treatment (10 mg/kg), esophageal neotissues substantially downregulated p-mTOR expression in respect to vehicle controls (Fig. 6A). We next observed that pharmacologic inhibition of PI3K or Akt activation via NVP-BEZ235 and GSK690693, respectively led to significant reductions in pan-CK + epithelia in neotissues compared to corresponding vehicle-treated controls (Fig. 6B–D). IHC and histomorphometric evaluations demonstrated that the percentage of Ki67 + CK + epithelial cells present at the anastomotic boundaries of both inhibitor groups tested was not significantly attenuated relative to parallel control levels (Fig. 6e,f). Interestingly, dual administration of Z-VAD-fmk with PI3K or Akt antagonists partially rescued the loss of pan-CK + epithelia in remodeling tissues (Fig. 6B–D). These data demonstrate that PI3K and Akt activation regulate epithelial wound healing partly through caspase inhibition.

**Figure 7:**
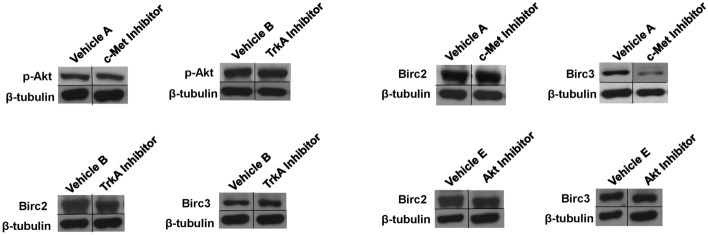
HGF/c-MET and NGF/TrkA signaling pathways influence esophageal epithelial regeneration in an Akt independent manner and in some cases modulate the expression of the anti-apoptotic protein, Birc3. Immunoblot analyses of p-Akt, Birc2, Birc3, and β-tubulin protein expression levels from neotissues treated with vehicle or inhibitors for 7 days of scaffold implantation. Vehicle and inhibitor-treated comparisons were made on the same immunoblots and with equal amounts of protein loaded per lane (N = 3–4 animals per group). Targeted bands were cropped from original full length immunoblots displayed in Supplementary Fig. S2.

We next investigated if c-MET and TrkA-mediated effects on epithelial regeneration were dependent on Akt activation (Fig. 7). Surprisingly, immunoblot analyses revealed no substantial change in p-Akt levels in esophageal tissues exposed to c-MET or TrkA antagonists compared to baseline. These data suggest that HGF/c-Met and NGF/TrkA signaling pathways govern pro-survival responses in esophageal epithelial progenitors in a manner independent of Akt activation. We then performed immunoblotting of other anti-apoptotic regulators, Birc2 and Birc3, in rats treated with c-MET, TrkA, or Akt inhibitors or vehicle controls given their known role as negative regulators of caspase function^26,27^. Our results demonstrated that the c-MET inhibitor substantially downregulated Birc3 protein expression in reconstructed esophageal tissues relative to the vehicle cohort while no effect on Birc2 was observed. In contrast, protein expression levels of Birc2 and Birc3 were not attenuated following treatment with TrkA or Akt inhibitors. These findings suggest that HGF/c-MET signaling may modulate pro-survival signals in the esophageal neotissues via Birc3 expression.

## Discussion

**Figure 8:**
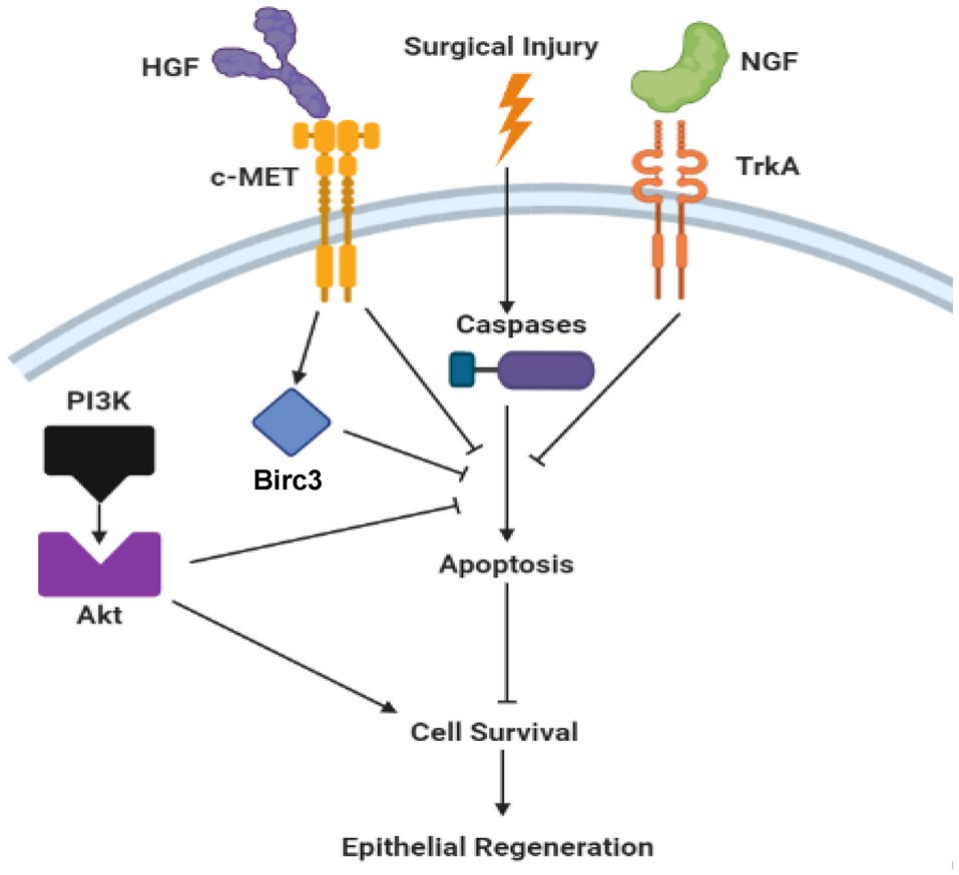
Model of signaling cascades which control epithelial regeneration during repair of surgical esophageal defects. Following surgical integration of acellular grafts, c-MET and TrkA receptors are activated in host epithelial cells buttressing the defect site via respective binding of HGF and NGF ligands. These pathways lead to inhibition of pan-caspase activity in the neoepithelium which mitigates apoptosis and encourages epithelial survival allowing for epithelial regeneration to occur. In addition, c-MET activation leads to upregulation of the anti-apoptotic protein, Birc3. In parallel, surgical injury also activates PI3K which leads to phosphorylation of Akt that is capable of exerting pro-survival stimuli in the neoepithelium partly through pan-caspase inhibition. Created with BioRender.com.

In present study, we established a comprehensive map of the molecular signaling dynamics which occur during regeneration of surgical defects repaired with acellular grafts. Quantitative proteomics and in silico analyses identified several cytokine signaling pathways with significant z-score activation during de novo epithelial formation. Pharmacological inhibition studies demonstrated that HGF/c-MET, NGF/TrkA, PI3K, and Akt signaling mechanisms all play significant roles in the epithelialization of neotissues. These molecular pathways were found to exert pro-survival signals all or in part through inhibition of caspase activity and/or Birc3 expression as modeled in Fig. 8. Although expression of caspases 1, 6 and 8 were increased in neotissues over NSC (Fig. 2B), we were unable to confirm which of these caspase(s) were regulated by our observed anti-apoptotic signaling cascades due to the lack of commercially-available primary antibodies necessary to detect active caspase subtypes in the neoepithelium alone. Nevertheless, HGF/c-MET and NGF/TrkA-dependent survival mechanisms may play key roles in esophageal epithelial regeneration by (1) protecting host epithelial progenitors from the acute inflammatory microenvironment as they populate graft sites and/or (2) serving to prevent cell death following loss of basement membrane attachment during stratification as seen during differentiation of other keratinocyte phenotypes^28^.

The nature of the epithelial regenerative response observed in our system appears to differ from other previously reported in vivo models of esophageal injury and repair both in terms of which pathways govern epithelialization and their function in the process. For instance, EGFR signaling has been shown to promote epithelial proliferation in the porcine esophagus following mucosal damage induced by sclerotherapy^15^, however our study demonstrated that inhibition of this pathway produced no significant effects on either mitogenic activity or de novo epithelial formation. In addition, published studies of epithelial wound healing in rats following caustic esophageal injury revealed HGF/c-MET signaling as potent regulator of epithelial proliferation^18^. In contrast, our results revealed a primarily pro-survival function of this molecular cascade with the absence of significant cell cycle effects. Taken together, these observations suggest that the mode of esophageal injury may play a key role in dictating selective repair mechanisms. This feature is reminiscent of urothelial regenerative processes wherein progenitor cellular hierarchy is modulated by the type of surgical damage encountered^29^. It should also be noted that growth factor dependency in esophageal regenerative processes may also be impacted by physiological differences in the species, sex, and age of the animal model deployed^30,31^.

Our data provide evidence that epithelial regeneration following onlay esophagoplasty is concurrently mediated by both Akt-dependent and -independent mechanisms which all or in part suppress caspase activity. Activated Akt is known to negatively regulate the initiator caspase 9 via phosphorylation of serine-196 which inhibits protease activity and dampens processing of downstream substrate caspases to mitigate apoptosis^32^. Stimulation of Akt activity has also been reported to promote caspase-independent, cell survival pathways through phosphorylation of various targets such as forkhead proteins, nuclear factor kappa-light-chain-enhancer of activated B cells (NF-kB), and BCL2 associated agonist of cell death (BAD) protein^33–35^. Future examinations are required to establish if these Akt-dependent, pro-survival cascades play a role in de novo epithelial formation following esophageal surgical reconstruction. In addition, we also observed that Akt phosphorylation in neotissues is independent of c-MET and TrkA activation and therefore other upstream regulators are likely involved. In Cluster 1 of putative signaling pathways (Supplementary Fig. S1), we observed downregulation of PTEN (phosphatase and tensin homolog) signaling relative to NSC controls following scaffold implantation and throughout neotissue formation. PTEN is a lipid phosphatase that, through dephosphorylation of the second messenger phosphatidylinositol 3,4,5-trisphosphate (PtdInsP3), inhibits PI3K signaling^36,37^. Therefore, we hypothesize that downregulation of PTEN signaling after surgical injury leads to activation of PI3K and its subsequent target, Akt, sufficient to mediate propagation of pro-survival signals.

We also uncovered that NGF/TrkA and HGF/c-MET signaling can attenuate caspase function in an Akt-independent manner to promote constructive remodeling of the neoepithelium. How this process occurs is unknown, however our results show that c-MET activation is capable of inducing the expression of the inhibitor of apoptosis protein, Birc3 following esophageal reconstruction. Birc3 has been previously shown to indirectly regulate caspase activation through E3 ligase activity, tumor necrosis factor (TNF)-signaling and nuclear factor kappa B (NFkB) signaling^38^ and therefore may play a role in neoepithelial survival. In addition, published studies in neuronal cell lines have shown that NGF-mediated inhibition of apoptosis can occur in an Akt-independent manner by removal of active caspase-3 via lysosomes^39^. c-MET also has the ability to block execution of apoptosis via a tandem pair of caspase-3 cleavage sites located on the cytoplasmic tail which bait, trap, and disable the active site of the caspase-3 without the involvement of Akt^40^. Further study is warranted to ascertain if these Akt-independent mechanisms contribute to esophageal epithelial regenerative processes.

Pharmacologic inhibition of caspase activity has emerged as a potential therapeutic strategy for enhancing tissue regeneration. Matsui and colleagues demonstrated that direct infusion of a pan-caspase inhibitor into the ear promotes the survival of hair cells and protects against vestibular functional deficits after aminoglycoside treatment^41^. Similarly, a study by Zhao et al. revealed that intranasal delivery of a caspase 1 inhibitor led to neuroprotective effects and improvement in functional neurologic recovery following transient global cerebral ischemia^42^. In addition, pan-caspase inhibition has also been shown to enhance skeletal muscle repair after crush injury of the soleus muscle in rats^43^. Our results suggest that the deployment of caspase inhibitors to encourage esophageal tissue regeneration may be a promising therapeutic approach in cases wherein epithelial formation is impeded following surgical reconstruction.

Preclinical investigations of acellular biomaterials for reconstruction of long-gap, tubular esophageal defects often show deficiencies in the ability of tissue engineered grafts to support skeletal muscle formation as well as de novo vascularization and innervation of neotissues^44,45^. A better understanding of signal transduction pathways which govern myogenesis, neurogenesis, and vascularization during healing of patch defects as described in the current study may inform the development of instructive biomaterial designs which can improve tubular neotissue maturation via controlled release of selective pathway agonists. De novo vascularization and innervation processes in semi-circumferential, rat esophageal neotissues have been previously reported to peak 7 days following BLSF scaffold implantation^46^. Therefore, pro-vascular signaling pathways identified in Clusters 3 and 4 such as PDGF and EGF signaling^47,48^ may be likely candidates for mediating vascular remodeling in neotissues, while the neurogenic effector, NGF/TrkA signaling^49^ may be an important regulator of neotissue innervation. Since skeletal muscle formation peaks in esophageal neotissues between 1 and 2 months post-operatively in our surgical model^46^, pro-myogenic pathways with similar kinetics found in Clusters 5–7 such as sphingosine-1-phosphate signaling^50^ and ephrin receptor signaling^51^ may be involved in myogenic differentiation. Future studies will investigate the role these pathways play in esophageal tissue regeneration.

In conclusion, we have characterized the molecular landscape of the esophageal repair process following surgical reconstruction with acellular grafts and have identified NGF/TrkA, HGF/c-MET, PI3K, and Akt signaling cascades as significant regulators of de novo epithelial formation. Activation of these pathways predominantly provides pro-survival stimuli via Akt-dependent and -independent mechanisms of caspase inhibition which allow host epithelial progenitors to reconstitute the epithelial barrier. These results shed light on the molecular machinery governing scaffold-mediated, constructive remodeling of esophageal neotissues and may lead to advancements in regenerative medicine strategies for gastrointestinal diseases.

## Methods

### Onlay rat esophagoplasty model

All animal experiments were performed under protocol 16-05-3161R following approval by the Boston Children’s Hospital Animal Care and Use Committee. All animal manipulations were carried out following the recommendations from the National Institutes of Health guide for the care and use of laboratory animals. In addition, this study was carried out in compliance with the ARRIVE guidelines (https://arriveguidelines.org). Male Sprague–Dawley rats (8–9 weeks of age, ~ 300–350 g, Charles River Laboratories, Wilmington, MA) were randomized and subjected to onlay esophagoplasty with bi-layer SF (BLSF) grafts as previously described^46^. Briefly, rats were maintained on a nutritionally balanced liquid diet formulation (TestDiet, Richmond, IN mixed with PediaSure, Abbott Laboratories, Columbus, OH) for 1 day prior to surgery and 3 days post-operatively. Animals were also given to free access water during the study period. General anesthesia was induced and maintained in animals by isoflurane inhalation. Under sterile conditions, the lower esophagus was exteriorized by hanging with two vessel loops through an upper midline laparotomy incision. A 7 × 3 mm^2^ elliptical defect was fashioned in the anterior esophagus 5 mm above the esophagogastric junction by surgical tissue resection. An elliptical BLSF graft of equal size was anastomosed into the defect site with running 7-0 polyglactin sutures. Four 7-0 nonabsorbable polypropylene sutures were placed at the proximal/distal and lateral edges of the graft in order to identify the original implantation region. The matrix was then covered with an omental patch and the esophagus was placed back into the abdomen and tissue layers were sutured closed. Immediately after surgical reconstruction, the abdominal incision was injected with 0.5% bupivacaine diluted 1:1 with saline as well as Buprenorphine SR (1.2 mg/kg, subcutaneously) for post-operative pain management. In addition, rats also received meloxicam (1 mg/kg, subcutaneously) for 3 days post-operatively. All animals were transferred from liquid diet to standard rat chow 3 days after surgical manipulations and were weighed weekly until scheduled euthanasia by carbon dioxide asphyxiation.

For quantitative proteomic studies, rats implanted with BLSF grafts (N = 4 per timepoint) were sacrificed at 1 day, 1 week, 1 month, and 2 months post-repair. Using biopsy tissue punches, host esophageal tissues surrounding 2 mm of the original defect perimeter were harvested following 1 day of scaffold implantation, while neotissues in subsequent study timepoints were isolated from the initial graft region. Esophageal tissue specimens procured from 4 nonsurgical controls (NSC) were utilized as baseline controls. Parallel histological and immunohistochemical (IHC) evaluations were performed as described below on esophageal tissues from an additional 4 rats per study timepoint to characterize the stages of constructive remodeling.

For pharmacologic inhibitor experiments, rats were injected intraperitoneally with selective inhibitors to signaling cascades identified from proteomic analyses or equal amounts of corresponding vehicle solutions for 7 days following esophageal reconstruction with BLSF scaffolds. Animals were administered inhibitor or vehicle formulations 24 h prior to surgery, 1 h before onlay esophagoplasty, and daily for 1 week until harvest. The experimental design consisted of animals exposed to the following inhibitors alone or in combination: SU11274, c-MET inhibitor (6 mg/kg); erlotinib, epidermal growth factor receptor (EGFR) inhibitor (10 mg/kg); NVP-BEZ235, PI3K inhibitor (10 mg/kg); GSK690693, Akt inhibitor (10 mg/kg); GW441756, TrkA inhibitor (1.7 mg/kg); crenolanib, platelet-derived growth factor receptor (PDGFR) inhibitor (10 mg/kg); and carbobenzoxy-valyl-alanyl-aspartyl-[Omethyl]-fluoromethylketone (Z-VAD-fmk), pan-caspase inhibitor (5 mg/kg). Vehicles for control animals included: Vehicle A for SU11274, 30% polyethylene glycol (PEG) 400 + 0.5% Tween 80 + 5% Propylene in distilled water; Vehicle B for GW441756 and U0126, 10% dimethylsulfoxide (DMSO) + 50% PEG 300 in distilled water; Vehicle C for erlotinib, 5% DMSO + 45% PEG 300 in distilled water; Vehicle D for crenolanib and NVP-BEZ235, 10% N-Methyl-2-pyrrolidone (NMP) in 90% PEG 300; Vehicle E for GSK690693, 5% DMSO + 30% PEG 300 in distilled water, and Vehicle F for Z-VAD-fmk, 100% DMSO. Following animal euthanasia, esophageal neotissues were evaluated by histological, IHC, and histomorphometric analyses or immunoblotting as detailed below.

### Quantitative proteomics

Tandem mass tagging (TMT)-based quantitative proteomics was performed essentially as previously described^52^. Briefly, protein was extracted from esophageal tissues using lysis buffer (80 mM Tris–HCl, 4% SDS, 100 mM DTT pH7.4) and thorough sonication in a water-bath sonicator (Elma S180H), followed by incubation at 95 °C for 5 min and centrifugation at 16,000×*g* for 10 min. The protein concentration was measured using the Pierce 660 nm Assay Kit (Thermo Scientific) according to the manufacturer’s instruction. From each sample, 60 μg of protein was digested with trypsin using filter-aided sample preparation (FASP)^53^ and labeled with TMT10plex reagents in parallel. Subsequently, each set of TMT10plex-labeled peptides were merged, desalted with C_18_ spin columns (Thermo Scientific), and fractionated via high-pH reverse phase liquid chromatography (RPLC) using an Ultimate 3000 XRS system (Thermo Scientific). For high-pH RPLC, about 50 μg TMT-labeled peptides were loaded onto a 100-mm Hypersil GOLD C_18_ column (2.1 mm inner diameter, 3 μm particle size, 175 Å pore size) (Thermo Scientific), flushed for 3 min with solvent A (10 mM ammonium formate, pH 10), and then separated with a 7-min linear gradient of 0–40% B (10 mM ammonium formate, 95% acetonitrile, pH 10.0). A total of 24 fractions were collected, concatenated into 12 fractions, and dried down in a SpeedVac (Thermo Scientific).

Tryptic peptides in each high-pH RPLC fraction were redissolved with 0.2% formic acid, followed by liquid chromatography-tandem mass spectrometry (LC–MS/MS) analysis using an EASY-nLC 1000 ultraperformance liquid chromatography system connected to an LTQ Orbitrap Elite Mass Spectrometer (Thermo Scientific), essentially as previously described^54^. Briefly, peptides were loaded onto a 2-cm trap column and separated by a 50-cm EASY-Spray column (Thermo Scientific, #ES803) heated to 55 °C. For low-pH RPLC separation, the mobile phases A and B consisted of 0.1% formic acid in water and in acetonitrile, respectively. The LC gradient was 4–24% B (150 min), 24–50% B (10 min), and 50–100% B (5 min) at a flow rate of 150 nL/min, followed by 100% B (15 min) at a flow rate of 300 nL/min. Mass spectra were acquired in the data-dependent mode, and up to 15 most abundant precursor ions were selected for higher-energy collisional dissociation (HCD). The mass resolution was set as 120,000 for precursor ions and 60,000 for fragment ions. The isolation width and the normalized collision energy were set as 1.5 and 40, respectively.

Database searching was performed by Proteome Discoverer (v2.1) (Thermo Scientific), using the SEQUEST algorithm and previously described methods^55^. The acquired raw data were searched against the rat Uniprot protein sequence database, which was released on 04/14/2016 and contained 21,328 protein sequences. Database searching parameters were set as follows: (a) trypsin, up to two missed cleavage, (b) precursor ion tolerance of 10 ppm and fragment ion tolerance of 0.02 Da, (c) fixed modifications include carbamidomethylation of cysteines and TMT6plex modification of lysines and peptide N-term, and (d) variable modifications include acetylation of protein N-term, oxidation of methionine and deamidation of asparagines and glutamines. A standard false discovery rate (FDR) of 1% was applied to filter peptide-spectrum matches (PSMs), peptide identifications, and protein identifications. For protein quantification, to minimize erroneous quantification caused by precursor ion interference, peptides with > 30% precursor ion interference were excluded. For data normalization, “total peptide amount” was used. For data scaling, “on control channels” was adopted. Statistical analysis was performed using Perseus (v1.5.5.3) p values were calculated by two-tailed Student’s t test and corrected for multiple testing using the Benjamini–Hochberg method^55^. Gene Ontology (GO) enrichment analysis was performed on protein species regulated by twofold (log2ratio > 1 or <  − 1) in at least one reconstructed group (1 day, 1 week, 1 month, 2 months post-op) compared with NSC controls using the rat UniProt database from above to identify biological functions relevant to tissue regenerative processes in corresponding genes. To determine differentially expressed proteins (DEPs), only the proteins quantified across all the 20 samples were analyzed and those with q-values of < 0.05 and log2-transformed fold changes of > 0.348 in absolute value were accepted. Here, the cutoff of 0.348 was computed based on the normal distribution of all log2-ratios and corresponds to p = 0.05.

### Prediction of significantly regulated canonical pathways

Ingenuity Pathway Analysis (IPA; Qiagen, Redwood City, CA) was applied to identify significantly enriched canonical pathways. DEPs were fed to the software to calculate the pathway activity (z-score) and determine whether the activity of canonical pathways is increased or reduced at each time point. The overall activation/inhibition states of canonical pathways were predicted on the basis of a z-score algorithm. The significance for the canonical pathways was calculated by Fisher's exact test, right-tailed. The –log10 of p-value was used to perform comparative analysis to detect significantly regulated pathways. In total 340 pathways were predicted and filtering criteria (p-value < 0.05 and regulation of at least one > 2 or <  − 2 z-score in one time point) were chosen to filter significantly regulated pathways. Pathways with no activity prediction were disregarded due to the high clustering occurrence. Unsupervised clustering and heatmap generation were conducted using pheatmap (v1.0.12) package in the environment of RStudio (v.1.3.1093) (http://www.rstudio.com/).

### Gene clouds

To condense and visualize large amounts of protein enrichment data from a pathway analysis data set and find biological patterns, a cloud was generated with Wordle.net and Word cloud R package. The font size of a protein (tag) is determined by its incidence in the pathway analysis data set and the color is relevant to the type of protein.

### Prediction of the biological function of canonical pathways

We developed an R package “BioFun” that quantifies the involvement of each IPA canonical pathway in Biological Function Classification Database of IPA known as ingenuity canonical pathway^56^. The number of pathways in each cluster with a specific biological function were determined and shown as radar graphs.

### Immunoblotting

Following 7 days of scaffold implantation, neotissues from vehicle or inhibitor-treated rats (N = 3–4 per group) as well as NSC were homogenized and placed in cell lysis buffer (Cell Signaling, Danvers, MA) with protease inhibitor (Thermo Scientific) and 0.1% sodium dodecyl sulfate (SDS). Tissues were homogenized using FastPrep system (MP Biomedical, Irvine, CA). Protein content was determined using a MicroBCA protein assay (Thermo Scientific) and Bradford method. Samples were denatured in β-mercaptethanol-containing sample buffer. Polyacrylamide SDS (10%) gels were loaded with 15–40 μg of protein based and run for ~ 100 min at 100 V (V) in standard Tris Glycine buffer. The gels were transferred to nitrocellulose membrane overnight in tris–glycine buffer with methanol at 50 V. Membranes were blocked for 1 h in PBST with 0.1% BSA, and incubated overnight with primary antibodies for either phosphorylated Akt (p-Akt) [Cell Signaling, 1:1000 dilution], phosphorylated EGFR (p-EGFR) [Cell Signaling, 1:1000 dilution], phosphorylated TrkA (p-TrkA) [Cell Signaling, 1:1000 dilution], phosphorylated c-MET (p-cMET) [Cell Signaling, 1:1000], phosphorylated PDGFR (p-PDGFR) [Abcam, 1:1000 dilution], phosphorylated mammalian target of rapamycin (p-mTOR) [Abcam, 1:1000 dilution], baculoviral IAP repeat-containing protein 2 (Birc2) [Abcam, 1:500 dilution], baculoviral IAP repeat-containing protein 3 (Birc3) [Abcam, 1:500 dilution], β-tubulin (Cell Signaling, 1:1000) at 4 °C. Following incubation with species-matched, secondary antibodies conjugated to horseradish peroxidase (1:7500 dilution), proteins were detected by enhanced chemiluminescence using SuperSignal West Pico reagent (Thermo Scientific) and visualized on a Chemidoc XRS (BioRad, Hercules, CA). Images without saturation of signal were acquired using ImageJ software (version 1.47). Full length immunoblots are displayed in Supplementary Fig. S2.

### Histological, IHC, and histomorphometric analyses

Reconstructed esophageal conduits from experimental cohorts as well as control specimens were fixed in 10% formalin for 12 h, dehydrated in graded alcohols, and embedded in paraffin. Tissue section (5 µm) were stained with Masson’s trichrome using routine protocols. IHC analyses were performed on parallel sections using primary antibodies to the following makers as previously reported^9,46^: anti-Ki67 [Abcam, 1:200 dilution], anti-pan-cytokeratin (CK) [Dako, Carpinteria, CA, 1:150 dilution], anti-CK5 [Abcam, 1:200 dilution], anti-filaggrin (FG) [Abcam, 1:200 dilution], EGFR [Abcam, 1:250 dilution], PDGFR [Cell Signaling, 1:100 dilution], c-MET [Abcam, 1:25 dilution], TrkA [Santa Cruz Biotechnology, Dallas, TX, 1:50 dilution], nerve growth factor (NGF) [Abcam, 1:250 dilution], and hepatocyte growth factor (HGF) [Abcam, 1:200 dilution]. For immunofluorescence, specimens were then incubated with species-matched Alexa Fluor 488, 594, and 647-conjugated secondary antibodies (Thermo Fisher Scientific) and nuclear counterstain was performed with 4′, 6-diamidino-2-phenyllindole (DAPI). For HGF and NGF detection, samples were incubated with species-matched horseradish peroxidase (HRP)-conjugated secondary antibodies in combination with hematoxylin counterstain. An Axioplan-2 microscope (Carl Zeiss MicroImaging, Thornwood, NY) was deployed for sample visualization and representative fields were acquired with Axiovision software (version 4.8).

Histomorphometric evaluations of neoepithelial regeneration in untreated, vehicle, and inhibitor-treated cohorts (N = 4–8 rats per group) were carried out using previously reported methods^46,57^. Neotissues boundaries were discerned from host esophagus tissues through identification of nonabsorbable marking sutures placed at the anastomotic periphery at the time of scaffold implantation. Image thresholding and area measurements were acquired with ImageJ software (version 1.47). Four independent microscopic fields (20× magnification) equally dispersed along the original graft site in pan-CK stained neotissues were used to calculate the percentage of stained tissue area per total tissue area investigated. In addition, the percentage of proliferating Ki67 + pan-CK + epithelial cells relative to the total pan-CK + population was also determined in 2 independent microscopic fields located at each side of the graft-host interface using similar methods. Multi-group (> 3 groups) comparisons of quantitative measurements between vehicle and inhibitor-treated groups were executed with the Kruskal–Wallis test in combination with post hoc Dunn’s test for pairwise comparisons. Two group comparisons were performed with the Mann–Whitney U test. p < 0.05 was considered significant for all described statistical tests.

## Supplementary Information

Supplementary Information 1.

Supplementary Information 2.

## Data Availability

The raw/processed data required to reproduce these findings will be shared upon request.
