# Supplementary material for: Molecular mechanisms of esophageal epithelial regeneration following repair of surgical defects with acellular silk fibroin grafts
Source: Sci Rep. 2021 Mar 29;11:7086. doi: 10.1038/s41598-021-86511-9 (PMC8007829; doi:10.1038/s41598-021-86511-9)

**Molecular Mechanisms of Esophageal Epithelial Regeneration Following Repair of Surgical Defects with Acellular Silk Fibroin Grafts**

Gokhan Gundogdu, Mehmet Tosun, Duncan Morhardt, Ali Hashemi Gheinani, Khalid Algarrahi, Xuehui Yang, Kyle Costa, Cinthia Galvez Alegria, Rosalyn M. Adam, Wei Yang, Joshua R. Mauney

**Supplementary Figure S1. Pathway signaling modulation in supplementary pathways identified by unbiased clustering in rat esophageal tissues following reconstruction with acellular grafts.** [**A-E**] Line charts of signaling cascades in each cluster based on z-score activation encountered over the course of esophageal healing. Data are represented of Clusters 1, 2, 5-7 detailed in Figure 2A. N= 4 animals were analyzed per experimental condition.

**
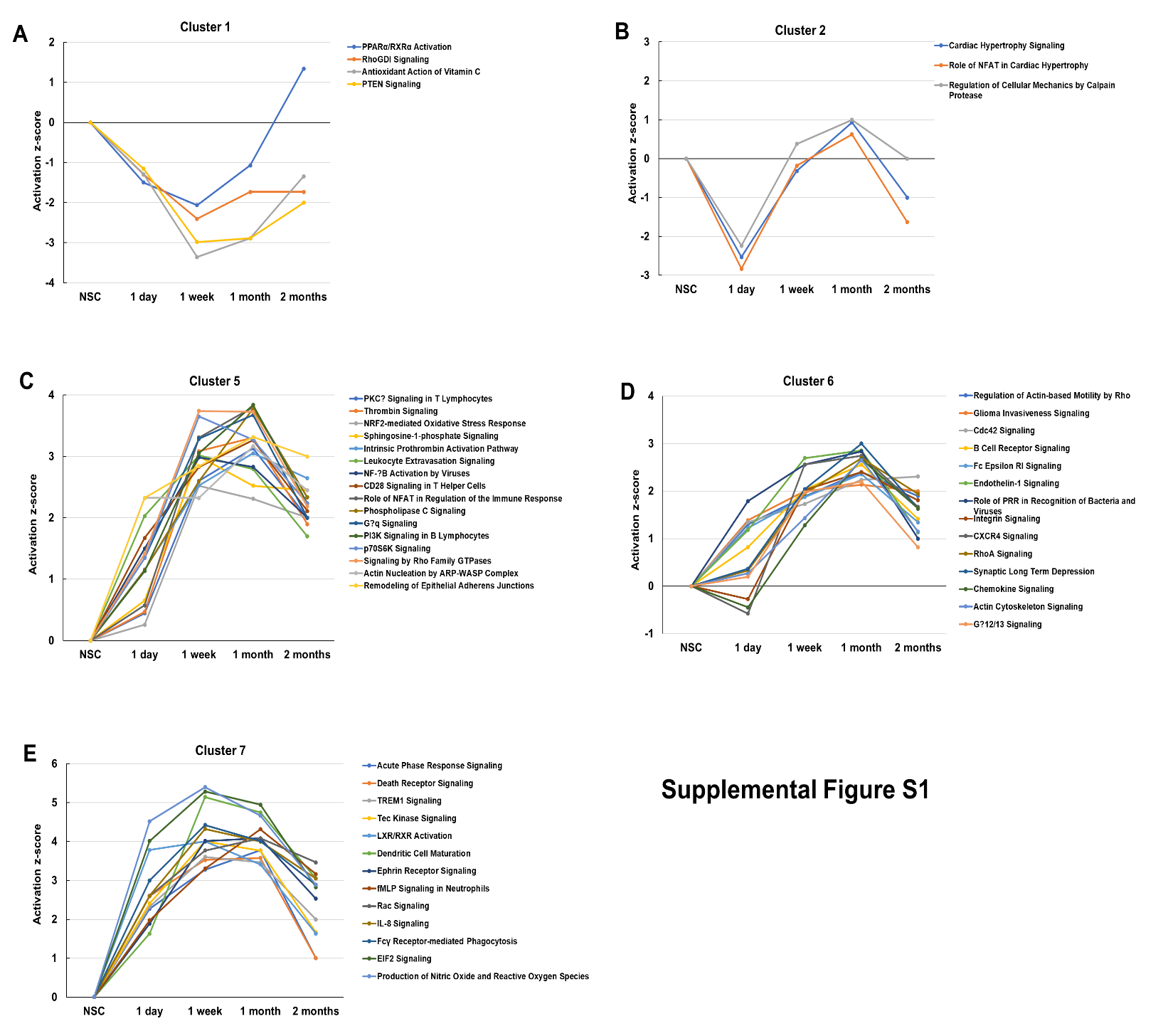
**

**Supplementary Figure S2. Full Length Immunoblots from Data Represented in Figures 5-7.** [**A-D**] Immunoblot analyses of p-cMET, p-TrkA, p-EGFR, p-PDGFR and β-tubulin protein levels from neotissues treated with vehicle or inhibitors for 7 days of scaffold implantation detailed in Figure 5A. [**E**] Immunoblot analysis of Akt target, p-mTOR and β-tubulin protein levels from neotissues treated with vehicle or Akt inhibitor for 7 days of scaffold implantation displayed in Figure 6A. [**F-M**] Immunoblot analyses of p-Akt, Birc2, Birc3, and β-tubulin protein expression levels from neotissues treated with vehicle or inhibitors for 7 days of scaffold implantation displayed in Figure 7. For all panels, vehicle and inhibitor comparisons were made on the same blots and with equal amounts of protein loaded per lane. Selected lanes detail representative data from N=3-4 animals per group in all panels. Bands of interest are boxed in red.


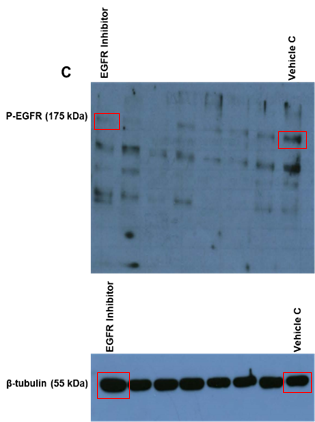

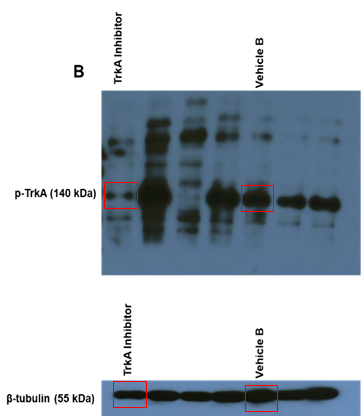

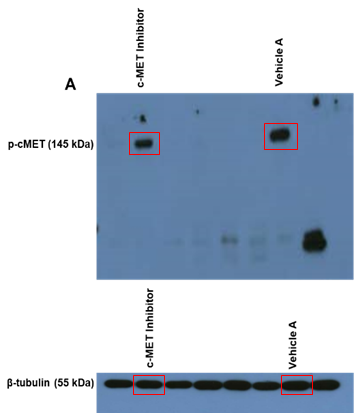


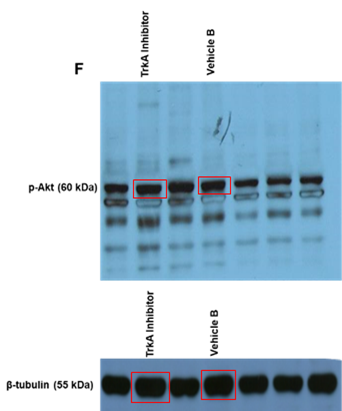

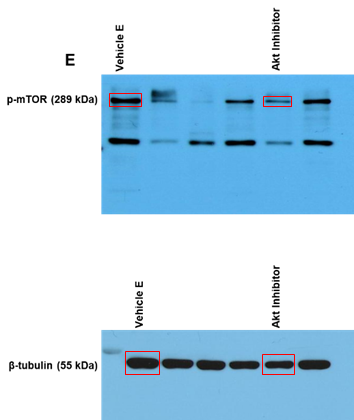

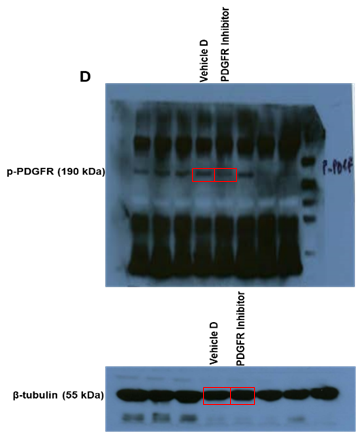


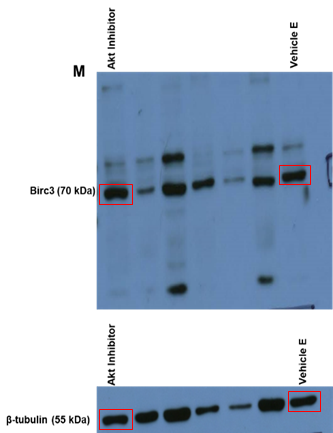

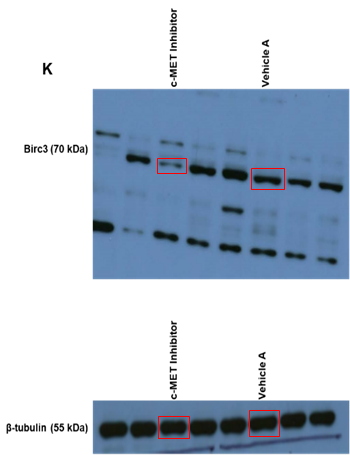

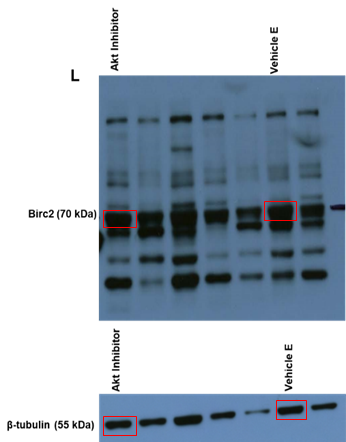

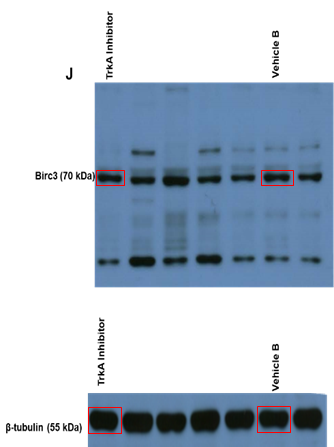

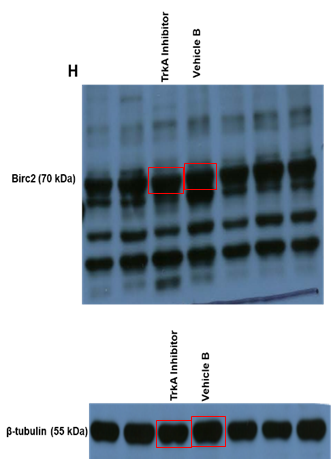

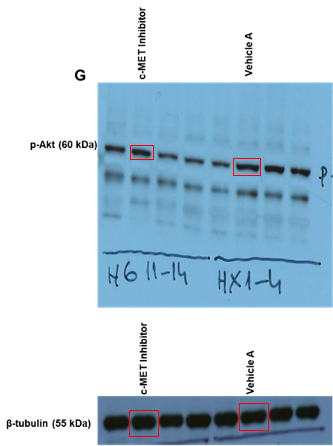


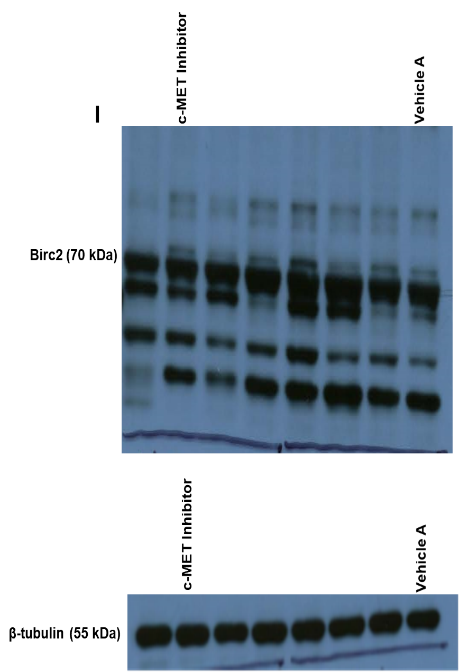

Supplement: Supplementary file 1 — Supplementary Information 1. [file 41598_2021_86511_MOESM1_ESM.docx]
